# The role of narrative medicine in cultivating professional competencies: a mediation analysis of empathy among medical students

**DOI:** 10.1186/s12909-026-09183-x

**Published:** 2026-04-11

**Authors:** Weijing Liu, Rixiang Xu, Hongli Ji, Yamin Liu, Chengyang Hu

**Affiliations:** 1 Department of Foreign Languages, School of Medical Humanities, Anhui Medical University, 81 Meishan Road, Hefei, 230032 China; 2 Department of Humanistic Medicine, School of Medical Humanities, Anhui Medical University, 81 Meishan Road, Hefei, 230032 China; 3 Department of Epidemiology and Biostatistics, School of Public Health, Anhui Medical University, 81 Meishan Road, Hefei, 230032 China

**Keywords:** Narrative medicine, Medical education, Empathy, Professional identity, Humanistic care, Mediation analysis, Medical students

## Abstract

**Introduction:**

Narrative medicine has emerged as a promising approach to enhance medical students’ professional competencies, but how it works remains unclear. Understanding whether and how narrative medicine influences professional identity, humanistic care, and empathy is crucial for medical education reform. This study investigated the associations of narrative medicine competencies with three key professional outcomes (professional identity, humanistic care, and empathy), with particular attention to the mediating role of empathy.

**Methods:**

A cross-sectional survey was performed among 594 medical students in China. Participants completed validated instruments assessing narrative medicine knowledge, narrative literacy, professional identity, humanistic care, and empathy. Multiple linear regression and bootstrap mediation analyses were performed to examine direct and indirect effects.

**Results:**

Narrative medicine knowledge and narrative literacy are associated with all outcomes. Empathy significantly mediated the relationships between narrative medicine competencies and professional outcomes. For narrative medicine knowledge, empathy accounted for 56.1% (95% CI [43.6%, 71.0%]) of the total effect on professional identity and 61.0% (95% CI [50.9%, 72.0%]) on humanistic care. For narrative literacy, the mediation proportions were 44.1% (95% CI [30.9%, 59.0%]) and 54.1% (95% CI [42.7%, 65.0%]), respectively.

**Conclusions:**

Empathy statistically mediates the associations between narrative medicine competencies and professional development outcomes. Narrative literacy is associated with professional outcomes through both empathy-mediated and direct pathways, while narrative medicine knowledge operates primarily through empathy. These findings suggest that medical education should prioritize hands-on narrative practices while retaining theoretical teaching to optimize professional development.

## Introduction

The cultivation of humanistic qualities in medical students has become increasingly recognized as essential to addressing contemporary healthcare challenges, including burnout, depersonalization, and declining patient satisfaction [1, 2]. Medical educators worldwide have acknowledged that technical proficiency alone is insufficient for delivering compassionate, patient-centered care [3, 4]. Professional identity formation, humanistic care capacity, and clinical empathy represent core competencies that distinguish excellent physicians from merely competent ones [5, 6].

Narrative medicine, pioneered by Rita Charon, offers a theoretical and practical framework for developing these humanistic competencies [7]. Defined as “medicine practiced with the narrative competence to recognize, absorb, interpret, and be moved by the stories of illness” [8], narrative medicine has been integrated into medical curricula globally with promising results [9–11]. Yet much of the prior work has focused on showing that narrative interventions work, rather than explaining why or how they produce their effects [12, 13].

Previous studies have distinguished between narrative medicine knowledge (theoretical understanding of narrative medicine concepts, illness narratives, and the foundation of narrative medicine) and narrative literacy (practical competencies in reading, writing, and interpreting narratives in clinical contexts) [14, 15]. This distinction is critical because it reflects the difference between knowing about narrative medicine and being able to apply it in practice. Yet few studies have systematically compared how these two dimensions differentially influence medical students’ professional development. Understanding this distinction has important pedagogical implications. If narrative medicine knowledge and narrative literacy operate through different mechanisms, medical educators need to design curricula that address both dimensions appropriately rather than assuming that theoretical instruction alone will translate into practical competencies [16].

Empathy, the capacity to understand and share another’s emotional state while maintaining self-other distinction [17], has been proposed as a central mechanism linking narrative engagement to improved clinical outcomes [18, 19]. Theoretically, engaging with illness narratives enhances perspective-taking abilities, thereby fostering empathic understanding, which in turn promotes compassionate care and a strong professional identity [8, 20]. Despite this theoretical foundation, empirical evidence for empathy’s mediating role remains limited. Most studies have examined bivariate relationships between narrative interventions and empathy [21, 22] or between empathy and other outcomes [23, 24], but few have tested empathy as a mediator in a comprehensive pathway model. This lack of mechanistic evidence limits our ability to design more effective teaching. Moreover, the decline in empathy during medical training, a well-documented phenomenon [25, 26], makes identifying protective factors particularly urgent. If narrative medicine competencies help preserve or boost empathy, and empathy then drives professional growth, this would provide strong justification for embedding narrative medicine in core training.

Professional identity, the integration of personal and professional selves grounded in values, commitments, and sense of purpose [27], develops through socialization, role modeling, and reflective practice [28]. Narrative medicine may facilitate this development by helping students construct coherent narratives about their evolving professional selves [29]. However, whether this process operates directly or through enhanced empathic capacities remains unclear. Similarly, humanistic care capacity, the ability to recognize patients’ dignity, practice patient-centered communication, and maintain compassion under pressure [30], represents a critical outcome of medical education. While narrative medicine aims to cultivate humanistic orientations [31], the pathways from narrative engagement to humanistic practice still need clearer empirical mapping. The current literature reveals several key limitations including many studies use only pre-post designs and ignore natural differences in students’ narrative skills [32]; few distinguish between narrative medicine knowledge and narrative literacy as potentially distinct constructs with differing effects [33]; and mechanistic research testing mediating variables remains scarce, hindering theoretical advancement [34].

This study addresses these gaps by investigating the associations of narrative medicine knowledge and narrative literacy with professional outcomes, as well as the mediating role of empathy in the relationships between these narrative medicine competencies and professional outcomes.

## Methods

### Study design and participants

This cross-sectional study was conducted from September to October 2025 at Anhui Medical University, a comprehensive medical university in China. We employed convenience sampling to recruit undergraduate and graduate medical students. Inclusion criteria were: (1) currently enrolled medical students and (2) voluntary participation. Exclusion criteria included incomplete survey responses.

The study was approved by the Ethics Committee of Anhui Medical University (No. 81251030). All participants provided informed consent electronically before survey access. Sample size was calculated using G*Power 3.1 for multiple regression analysis with 15 predictors, effect size f2 = 0.15 (medium), α = 0.05, and power (1-β) = 0.95, yielding a minimum required sample of 139. We targeted at least 400 participants to guarantee sufficient power for the mediation analyses [35].

### Measures

#### Narrative medicine knowledge scale

We developed a 17-item scale assessing theoretical understanding of narrative medicine concepts, including the philosophical basis of narrative medicine, illness narratives, narrative ethics, and the role of stories in healing. Items were generated based on a comprehensive literature review and expert consultation, and the scale was piloted with 50 medical students and refined based on item analysis and expert feedback. Items used a 5-point Likert scale (1 = strongly disagree to 5 = strongly agree). Higher scores indicate greater narrative medicine knowledge. In this sample, Cronbach’s α = 0.87.

#### Narrative literacy scale

An 18-item instrument was independently developed to assess practical narrative medicine competencies including close reading, reflective writing, narrative interpretation, and clinical application of narrative skills, representing the applied dimension of narrative medicine that is conceptually distinct from theoretical knowledge. Items were generated through literature review, expert panel discussion, and student focus groups, and the scale was piloted alongside the Knowledge Scale to ensure discriminant validity. Response format was a 5-point Likert scale. Higher scores reflect greater narrative literacy. Cronbach’s α = 0.90.

#### Professional identity scale

We used the 27-item Chinese Medical Students’ Professional Identity Scale [36], which measures six dimensions: cognition (3 items), affect (5 items), commitment (4 items), behavior (6 items), expectation (5 items), and values (4 items). Total scores range from 27 to 135, with higher scores indicating stronger professional identity. Cronbach’s α = 0.93.

#### Humanistic care capacity scale

The Caring Ability Inventory (37 items) [37, 38] was adapted to measure medical students’ humanistic care capacity across three components: understanding (14 items), courage (13 items), and patience (10 items). After reverse-scoring 13 items (4, 8, 10, 11, 12, 13, 14, 16, 19, 26, 28, 29, 32), total scores range from 37 to 259. Higher scores indicate greater humanistic care capacity. Cronbach’s α = 0.91.

#### Jefferson scale of empathy - student version (JSE-S)

The validated Chinese version of the JSE-S [39] contains 20 items measuring empathy in healthcare contexts across dimensions: perspective-taking (10 items), compassionate care (8 items), and standing in the patient’s shoes (2 items). Ten items (1, 3, 6, 7, 8, 11, 12, 14, 18, 19) were reverse-scored. Total scores range from 20 to 140, with higher scores indicating greater empathy. Cronbach’s α = 0.88.

#### Demographic and educational variables

We collected data on sex, age, student status (undergraduate vs. graduate), major selection preference, family medical background, student leadership roles, humanities coursework, humanities awards, volunteer experience, self-rated interpersonal skills, self-rated empathy, and clinical internship experience.

### Data collection

The survey was administered electronically via the online platform Wenjuanxing (https://www.wjx.cn) from September to October 2025. Participants accessed the survey through a QR code distributed through student organizations and course announcements. The survey required approximately 10–15 min to complete. No compensation was provided. Data quality checks included attention-check items.

### Statistical analysis

Descriptive statistics (means, standard deviations, frequencies) were calculated for all variables. Normality was assessed using Shapiro-Wilk tests and visual inspection of Q-Q plots. For non-normally distributed data, Mann-Whitney U tests or Kruskal-Wallis tests were used for group comparisons; otherwise, independent t-tests or ANOVA were employed. Pearson correlations examined bivariate relationships among continuous variables. Multiple linear regression analyses tested the associations between narrative medicine competencies (knowledge and literacy) and each outcome (empathy, professional identity, humanistic care), with stepwise adjustment for demographic covariates.

Bootstrap mediation analyses (5,000 iterations) were conducted using the mediation package [40] to test whether empathy mediated the relationships between narrative medicine competencies and professional outcomes. Four mediation models were specified:

1. Narrative medicine knowledge → Empathy → Professional identity.
2. Narrative medicine knowledge → Empathy → Humanistic care.
3. Narrative literacy → Empathy → Professional identity.
4. Narrative literacy → Empathy → Humanistic care.

For each model, we estimated: (a) indirect effects (Average Causal Mediation Effect, ACME), (b) direct effects (Average Direct Effect, ADE), (c) total effects, and (d) proportion mediated. The 95% confidence intervals (CIs) were generated. The mediation models were theoretically driven, based on the conceptual framework that narrative medicine competencies may enhance empathy, which in turn may promote professional development. However, given the cross-sectional design, these analyses test statistical indirect effects and do not establish causal pathways.

All analyses were conducted using R version 4.3.1 (R Core Team, 2023). Statistical significance was set at α = 0.05 (two-tailed).

## Results

### Participant characteristics

A total of 697 medical students completed the survey, yielding 594 valid questionnaires. Table 1 presents demographic and educational characteristics. The sample comprised 279 males (47.0%) and 315 females (53.0%), with a mean age of 21.08 years. Undergraduate students constituted 83.8% (*n* = 498) and graduate students 16.2% (*n* = 96). Most participants (73.2%) reported that medicine was their first major choice. Over half (85.0%) had taken humanities courses, 29.1% had received student leadership roles, and 92.6% had volunteer experience. Most rated their interpersonal skills (76.6%) and empathy (48.0%) as good or very good.

**Table 1:** Demographic and educational characteristics of participants (*n* = 594)

Characteristic	*n* (%) or Mean ± SD
Sex	
Female	315 (53.0)
Male	279 (47.0)
Age	21.08 ± 2.58
Student status	
Undergraduate	498 (83.8)
Graduate	96 (16.2)
Urban/rural origin	
Urban	308 (51.9)
Rural	286 (48.1)
Only child	
Yes	215 (36.2)
No	379 (63.8)
Medicine as first choice	
Yes	435 (73.2)
No	159 (26.8)
Family medical background	
Yes	176 (29.6)
No	418 (70.4)
Student leadership	
Yes	173 (29.1)
No	421 (70.9)
Humanities coursework	
Yes	505 (85.0)
No	89 (15.0)
Humanities awards	
Yes	164 (27.6)
No	430 (72.4)
Volunteer experience	
Yes	550 (92.6)
No	44 (7.4)
Self-rated interpersonal skills	
Excellent	105 (17.7)
Good	455 (76.6)
Poor	34 (5.7)
Self-rated empathy	
High	285 (48.0)
Moderate	297 (50.0)
Low	12 (2.0)
Clinical internship	
Yes	380 (64.0)
No	214 (36.0)

*Abbreviation*: *SD* standard deviation

Female students scored significantly higher than males on narrative medicine knowledge, narrative literacy, and empathy. No significant sex differences emerged for professional identity or humanistic care. Students with leadership experience scored significantly higher on narrative literacy, professional identity, and humanistic care. Students with higher self-rated interpersonal skills and empathy demonstrated significantly higher scores across all study variables. No significant differences were found for student status, urban/rural origin, only-child status, first-choice major, family medical background, humanities coursework, or clinical internship experience (Table 2).

**Table 2:** Narrative medicine knowledge, narrative literacy, humanistic care, professional identify, and empathy scores stratified by demographic and educational characteristics (*n* = 594)

Characteristics	Group	Narrative medicine knowledge Mean (SD)	*P*-value	Narrative literacy Mean (SD)	*P*-value	Humanistic care Mean (SD)	*P*-value	Professional identity Mean (SD)	*P*-value	Empathy Mean (SD)	*P*-value
Sex	Female	50.2 (6.52)	0.007	71.5 (8.62)	0.050	179 (17.20)	0.142	107 (15.80)	0.875	111 (16.0)	< 0.001
	Male	48.7 (7.08)		69.8 (8.97)		177 (18.60)		106 (16.80)		106 (16.3)	
Student status	Undergraduate	49.4 (6.70)	0.436	70.8 (8.84)	0.828	177 (17.90)	0.330	106 (16.30)	0.917	108 (16.4)	0.746
	Graduate	50.0 (7.50)		70.6 (8.76)		180 (18.00)		107 (16.30)		109 (15.7)	
Urban/rural origin	Urban	49.1 (6.74)	0.286	70.7 (9.14)	0.797	178 (19.10)	0.832	106 (17.10)	0.536	107 (17.4)	0.280
	Rural	49.9 (6.91)		70.8 (8.48)		178 (16.60)		107 (15.40)		109 (15.0)	
Only child	Yes	49.5 (6.71)	0.763	71.4 (8.80)	0.299	179 (18.90)	0.434	107 (16.30)	0.196	108 (16.4)	0.911
	No	49.4 (6.90)		70.4 (8.82)		177 (17.30)		106 (16.30)		108 (16.3)	
Medicine as first choice	First	49.7 (6.93)	0.682	70.9 (9.06)	0.477	179 (18.50)	0.462	107 (16.20)	0.272	109 (16.6)	0.618
	Second	49.0 (5.98)		70.0 (7.70)		175 (16.10)		105 (16.80)		107 (15.1)	
	Third	48.5 (7.11)		70.7 (8.51)		177 (16.60)		103 (15.60)		108 (15.7)	
	Others	50.7 (6.36)		68.0 (8.89)		176 (14.90)		105 (17.80)		103 (17.4)	
Family medical background	Yes	49.5 (6.74)	0.727	70.5 (9.44)	0.982	178 (18.30)	0.869	107 (16.00)	0.635	108 (16.5)	0.556
	No	49.5 (6.87)		70.8 (8.56)		178 (17.80)		106 (16.40)		109 (16.2)	
Student leadership	Yes	50.3 (6.76)	0.071	72.2 (8.80)	0.007	181 (17.80)	0.004	109 (15.60)	0.010	110 (15.7)	0.180
	No	49.2 (6.84)		70.1 (8.77)		176 (17.80)		105 (16.40)		108 (16.5)	
Humanities coursework	Yes	49.6 (6.84)	0.157	70.7 (8.74)	0.802	178 (17.90)	0.574	106 (16.20)	0.154	108 (16.2)	0.411
	No	48.6 (6.70)		70.7 (9.34)		176 (17.90)		109 (16.40)		109 (16.8)	
Humanities awards	Yes	50.8 (6.86)	0.009	71.9 (9.37)	0.057	180 (18.40)	0.067	108 (16.20)	0.141	110 (16.2)	0.266
	No	49.0 (6.76)		70.3 (8.57)		177 (17.70)		106 (16.30)		108 (16.3)	
Volunteer experience	Yes	49.6 (6.79)	0.027	70.9 (8.72)	0.104	178 (17.80)	0.060	107 (15.90)	0.373	109 (16.0)	0.218
	No	47.2 (6.94)		68.6 (9.80)		174 (18.90)		104 (20.70)		105 (19.1)	
Self-rated interpersonal skills	Excellent	51.6 (7.66)	< 0.001	72.9 (9.67)	< 0.001	187 (19.60)	< 0.001	112 (16.20)	< 0.001	113 (15.9)	0.004
	Good	49.2 (6.49)		70.6 (8.49)		177 (16.60)		106 (15.70)		108 (16.2)	
	Poor	46.2 (6.81)		66.4 (8.76)		164 (16.70)		97.1 (18.90)		103 (16.8)	
Self-rated empathy	High	50.5 (6.77)	0.001	73.1 (8.41)	< 0.001	184 (17.10)	< 0.001	111 (15.10)	< 0.001	113 (15.1)	< 0.001
	Moderate	48.6 (6.75)		68.8 (8.61)		172 (16.20)		103 (15.60)		105 (16.3)	
	Low	46.4 (6.37)		63.3 (8.30)		163 (23.30)		93.7 (29.20)		100 (18.9)	
Clinical internship	Yes	49.8 (6.69)	0.161	70.8 (8.79)	0.652	178 (18.90)	0.810	106 (16.30)	0.152	108 (16.2)	0.913
	No	49.0 (7.05)		70.7 (8.89)		177 (16.00)		108 (16.20)		108 (16.5)	

*Abbreviation*: *SD* standard deviation

### Correlations among study variables

**Fig. 1:**
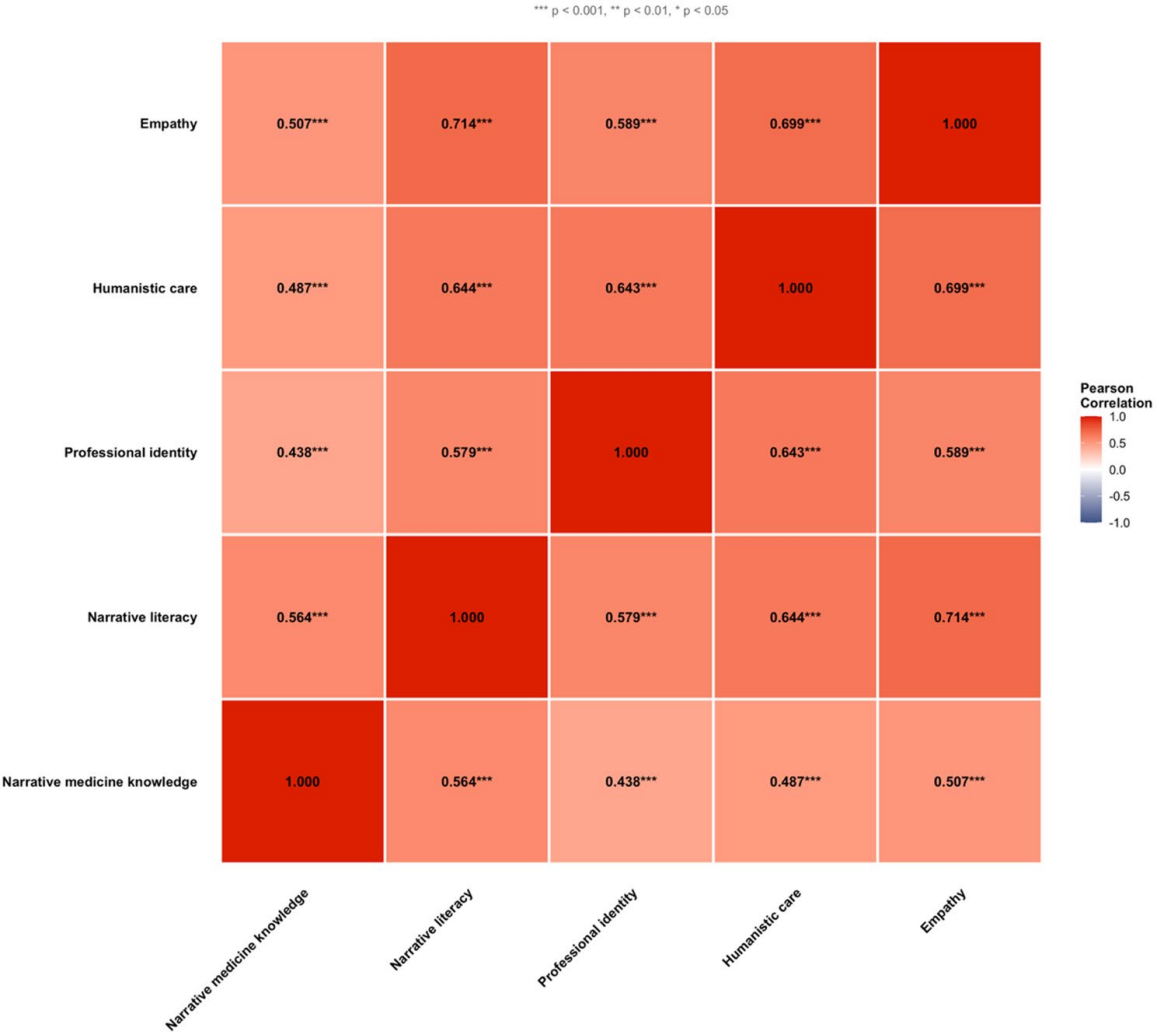
Correlation heatmap showing relationships among all five main study variables

Figure 1 presents Pearson correlation coefficients among the five main study variables. All correlations were positive and statistically significant (all *p* < 0.001). Narrative literacy showed the strongest correlations with empathy (*r* = 0.71), humanistic care (*r* = 0.64), and professional identity (*r* = 0.58). Narrative medicine knowledge demonstrated moderate correlations with all outcomes (*r* = 0.45–0.53). Empathy was strongly correlated with humanistic care (*r* = 0.66) and moderately correlated with professional identity (*r* = 0.51). The correlation between narrative medicine knowledge and narrative literacy was *r* = 0.56, indicating a moderate association consistent with related but distinct constructs. Multicollinearity diagnostics confirmed that Variance Inflation Factor (VIF) values for both predictors were below 2.0 in all mutually adjusted models, indicating stable coefficient estimates.

### Associations of narrative medicine knowledge and narrative literacy with empathy, professional identity, and humanistic care

Table 3 presents linear regression results predicting empathy, professional identity, and humanistic care. In the unadjusted models (Model 1), both narrative medicine knowledge and narrative literacy showed significant positive associations with all three outcomes. Narrative medicine knowledge was associated with humanistic care (β = 1.43, 95% CI: 1.22, 1.63), professional identity (β = 1.17, 95% CI: 0.97, 1.36), and empathy (β = 1.35, 95% CI: 1.17, 1.54). Similarly, narrative literacy demonstrated associations with humanistic care (β = 1.31, 95% CI: 1.18, 1.43), professional identity (β = 1.07, 95% CI: 0.95, 1.19), and empathy (β = 1.32, 95% CI: 1.21, 1.42). After mutual adjustment for both narrative medicine knowledge and narrative literacy (Model 2), the associations of narrative medicine knowledge with all outcomes were substantially attenuated but remained statistically significant. The fully adjusted models (Model 3), which controlled for sex and student status, yielded results nearly identical to Model 2, indicating that the associations held after accounting for these potential confounders.

**Table 3:** The associations of narrative medicine knowledge or narrative literacy with humanistic care, professional identity, and empathy (*n* = 594)

	Humanistic care			Professional identity			Empathy		
	Model 1	Model 2	Model 3	Model 1	Model 2	Model 3	Model 1	Model 2	Model 3
Narrative medicine knowledge	1.43 (1.22,1.63)	0.53 (0.32, 0.75)	0.53 (0.31, 0.74)	1.17 (0.97, 1.36)	0.44 (0.23, 0.64)	0.44 (0.23, 0.65)	1.35 (1.17, 1.54)	0.41 (0.23, 0.59)	0.40 (0.22, 0.57)
Narrative literacy	1.31 (1.18, 1.43)	1.10 (0.95, 1.25)	1.10 (0.96, 1.25)	1.07 (0.95, 1.19)	0.90 (0.75, 1.04)	0.90 (0.76, 1.05)	1.32 (1.21, 1.42)	1.16 (1.03, 1.28)	1.15 (1.03, 1.28)

Values represent unstandardized regression coefficients β (95% CI)

Model 1: Unadjusted (each predictor entered separately)

Model 2: Mutually adjusted for both narrative medicine knowledge and narrative literacy

Model 3: Fully adjusted for narrative medicine knowledge, narrative literacy, sex, and student status

### Mediation analysis

Bootstrap mediation analyses (5,000 iterations) were conducted to test whether empathy mediated the associations between narrative medicine competencies and professional outcomes. The results of the four mediation models are summarized in Table 4. For the pathway from narrative medicine knowledge to professional identity, empathy exerted a significant indirect effect (ACME = 0.61, 95% CI [0.49, 0.73], *p* < 0.001), accounting for 56.1% (95% CI: 43.6%, 71.0%) of the total effect. The direct effect remained significant (ADE = 0.47, 95% CI [0.27, 0.68], *p* < 0.001), indicating partial mediation. Similarly, for humanistic care, the indirect effect via empathy was significant (ACME = 0.82, 95% CI [0.69, 0.95], *p* < 0.001), explaining 61.0% (95% CI: 50.9%, 72.0%) of the total effect, with a significant direct effect (ADE = 0.52, 95% CI [0.34, 0.71], *p* < 0.001). Regarding narrative literacy, empathy significantly mediated the relationship with professional identity (ACME = 0.47, 95% CI [0.34, 0.62], *p* < 0.001), accounting for 44.1% (95% CI: 30.9%, 59.0%) of the total effect. The direct effect was also significant (ADE = 0.60, 95% CI [0.41, 0.79], *p* < 0.001), suggesting that narrative literacy influences professional identity through additional pathways independent of empathy. For humanistic care, the indirect effect was significant (ACME = 0.71, 95% CI [0.57, 0.84], *p* < 0.001), representing 54.1% (95% CI: 42.7%, 65.0%) of the total effect, with a significant direct effect (ADE = 0.60, 95% CI [0.43, 0.79], *p* < 0.001).

**Table 4:** The mediation effects of empathy in association of narrative medicine knowledge or narrative literacy with professional identity or humanistic care (*n* = 594)

Path	Indirect effect	Direct effect	Total effect	Proportion mediated (%)	*P*-value
A	0.61 (0.49, 0.73)	0.47 (0.27, 0.68)	1.08 (0.89, 1.26)	56.1 (43.6, 71.0)	<0.001
B	0.82 (0.69, 0.95)	0.52 (0.34, 0.71)	1.34 (1.16, 1.52)	61.0 (50.9, 72.0)	<0.001
C	0.47 (0.34, 0.62)	0.60 (0.41, 0.79)	1.07 (0.94, 1.20)	44.1 (30.9, 59.0)	<0.001
D	0.71 (0.57, 0.84)	0.60 (0.43, 0.79)	1.31 (1.18, 1.43)	54.1 (42.7, 65.0)	<0.001

A: Narrative medicine knowledge → Empathy → Professional identity

B: Narrative medicine knowledge → Empathy → Humanistic care

C: Narrative literacy → Empathy → Professional identity

D: Narrative literacy → Empathy → Humanistic care

Values represent unstandardized effect estimates (95% CI) from bootstrap mediation analyses with 5,000 iterations

Indirect effect = Average Causal Mediation Effect (ACME); Direct effect = Average Direct Effect (ADE)

Proportion mediated represents the percentage of the total effect explained by the indirect pathway through empathy

All models adjusted for sex and student status

## Discussion

This study offers solid support that empathy statistically mediates the associations between narrative medicine competencies and professional development outcomes in medical students. We found that narrative medicine knowledge and narrative literacy were associated with all outcomes of interest. Empathy significantly mediated these relationships, accounting for approximately 44–61% of total effects depending on the specific pathway, though these proportions should be interpreted with caution given the inherent uncertainty in the proportion mediated statistic.

This finding aligns with situated learning theory [41], which emphasizes that competencies develop through authentic practice rather than abstract instruction. Narrative literacy represents “knowing how” rather than merely “knowing that” [42], involving embodied skills that transform one’s stance toward patients and illness. For example, writing reflective narratives about real clinical encounters may build metacognitive awareness and perspective-taking in ways that simply reading about narrative medicine cannot achieve [43].

The practical implication is clear: medical curricula should give priority to experiential narrative activities. Rather than merely teaching students about the value of patient stories, educators should create structured opportunities for narrative engagement, patient narrative interviews, reflective writing assignments, close reading workshops, and narrative analysis of clinical cases [44, 45]. Our results indicate that such hands-on approaches will likely produce substantially greater benefits than purely didactic instruction.

The strong mediating role of empathy, especially in the pathway from narrative medicine knowledge to humanistic care (61% mediated), provides empirical support for theoretical models positing empathy as the mechanism linking narrative engagement to compassionate care [8, 18, 19]. This finding is noteworthy given the well-documented decline in empathy during medical training [25, 26]. Our data suggest that cultivating narrative medicine competencies may help protect against this erosion by maintaining students’ ability to take others’ perspectives and stay emotionally attuned.

The differential mediation patterns are theoretically informative. Narrative medicine knowledge appears to require empathy as a bridge to translate theoretical understanding into professional competencies. Simply knowing about narrative medicine does not directly strengthen professional identity or humanistic care; rather, such knowledge must foster empathic capacities, which then enable professional development. This sequential process may help explain why some narrative medicine courses yield limited practical impact if the teaching increases knowledge without also developing empathy. In contrast, narrative literacy showed substantial direct effects alongside mediated pathways, suggesting multiple mechanisms of influence. Literacy-based activities may directly develop communication skills, clinical reasoning, and reflective capacity independent of empathic enhancement. For instance, close reading can sharpen pattern recognition and interpretive sensitivity, while reflective writing can strengthen metacognitive monitoring, both valuable clinical skills that operate partly independently of empathy [46, 47].

The empathy decline during medical training represents a critical challenge for medical education [48]. Our findings suggest that narrative literacy may counteract this trend through a dual mechanism: directly maintaining empathic capacities through repeated perspective-taking practice, and indirectly supporting empathy by strengthening professional identity and humanistic values that motivate empathic engagement. Importantly, the strong correlation between empathy and humanistic care (*r* = 0.66) suggests that empathy is not merely a cognitive skill but is deeply embedded within broader humanistic orientations. Students who maintain high empathy likely do so because they have integrated humanistic values into their professional identities [49]. Narrative medicine may facilitate this integration by helping students construct coherent narratives about themselves as compassionate healers [29].

In this study, we found female students demonstrated significantly higher narrative medicine knowledge, narrative literacy, and empathy level. These findings are consistent with extensive research documenting female advantages in empathy and perspective-taking [50, 51]. Several explanations warrant consideration. First, socialization processes may encourage females to develop interpersonal sensitivity and emotional attunement from early childhood [52]. Second, females may be more drawn to and skilled at narrative activities given documented sex differences in reading preferences and verbal expression [53]. Third, medical culture may impose different expectations on males and females, with females experiencing greater pressure to demonstrate caring behaviors [54].

From an educational standpoint, these findings suggest that male students may benefit from additional support in developing narrative medicine competencies. Pedagogical approaches that resonate with diverse learning styles, including analytical, problem-solving approaches to narrative interpretation, may engage male students more effectively [55]. However, care must be taken to avoid reinforcing stereotypes or suggesting that empathy is inherently more difficult for males to develop.

Students with leadership experience showed higher narrative literacy, professional identity, and humanistic care. This pattern likely reflects bidirectional relationships: leadership roles may provide opportunities to develop interpersonal and reflective capacities, while students with strong professional identity may seek out leadership positions [56]. From a developmental perspective, leadership experiences offer authentic contexts for narrative sense-making, navigating interpersonal conflicts, reflecting on values, and constructing professional narratives [57].

The strong associations between self-rated interpersonal skills/empathy and all study variables merit careful interpretation. While some critics might dismiss self-ratings as mere self-perception bias, we believe they reflect genuine self-awareness. Students who recognize their own empathic capacities may be more likely to engage authentically in narrative activities, creating a virtuous cycle of development [58]. Alternatively, narrative engagement may enhance metacognitive awareness, leading to more accurate self-assessment.

Several limitations of the present study should be acknowledged. Most importantly, the cross-sectional design precludes definitive causal inference. While our theoretical model posits empathy as a mediator, it is plausible that students with a stronger pre-existing professional identity are more likely to develop narrative medicine competencies, which in turn reinforce empathy. Reciprocal relationships among these constructs are also possible. Longitudinal or experimental designs are needed to confirm the directionality of these relationships. Additionally, while the mediation effects were statistically robust, the relatively wide confidence intervals for the proportion mediated (e.g., 43.6%–71.0% for the narrative medicine knowledge to professional identity pathway) reflect inherent uncertainty in this statistic, which is common in mediation analyses. These proportions should be interpreted as approximate rather than precise estimates, and warrant confirmation in larger samples. In addition, reliance on self-reported measures may introduce biases such as social desirability and common method variance, highlighting the need for future studies to incorporate behavioral, physiological, and third-party assessments. The sample was limited to Chinese medical students, which may restrict generalizability to other cultural settings, particularly given differences between Western individualism and Chinese collectivism in relation to narrative practices. Furthermore, although the narrative medicine measures demonstrated good internal consistency, their validity requires further establishment, and the moderate correlation between narrative medicine knowledge and narrative literacy scales (*r* = 0.56) suggests some overlap, though multicollinearity diagnostics (VIF < 2.0) confirmed stable regression estimates. Potential moderators, such as personality traits, prior humanities exposure, or learning orientations, were not assessed, nor was the distinction between cognitive and affective empathy, which may have differing implications for clinical practice.

Despite these limitations, the findings offer several practical implications for medical education. Curricula should prioritize experiential narrative activities, such as reflective writing, close reading of patient narratives, and group discussions, over purely didactic approaches, integrating them throughout clinical training rather than isolating them in electives. Empathy should be positioned as an explicit learning objective, with clear links drawn between narrative engagement and its development. Providing structured scaffolding, faculty feedback, and peer-learning communities can enhance the impact on narrative competence, empathy, and professional identity formation. Longitudinal monitoring of empathy, sex-sensitive pedagogical strategies to engage diverse learners, and opportunities for student leaders to reflect narratively may further optimize outcomes.

## Conclusions

In conclusion, this study indicates that empathy partially mediates the associations between narrative medicine competencies and both professional identity and humanistic care among medical students, though the cross-sectional design precludes causal inference. Narrative literacy exerts influence through both empathy-mediated and direct pathways, while narrative medicine knowledge operates primarily through empathy. These findings highlight the value of prioritizing experiential activities, such as reflective writing, close reading, and narrative interpretation, in medical curricula to sustain empathy and promote humanistic professional development. As empathy decline remains a persistent challenge in medical training, narrative medicine emerges as a promising strategy to support professional formation. Future studies should adopt longitudinal and experimental designs to confirm the directionality of these relationships, explore moderators, and assess long-term effects on clinical practice and patient outcomes. Cross-cultural investigations are also needed to evaluate the generalizability of these mechanisms. 

## Data Availability

The datasets generated and analyzed during the current study are available from the corresponding author on reasonable request.
